# Epigenetic dysregulation in meningiomas

**DOI:** 10.1093/noajnl/vdac084

**Published:** 2022-06-06

**Authors:** Michelle A Wedemeyer, Ivo Muskens, Ben A Strickland, Oscar Aurelio, Vahan Martirosian, Joseph L Wiemels, Daniel J Weisenberger, Kai Wang, Debraj Mukerjee, Suhn K Rhie, Gabriel Zada

**Affiliations:** Department of Neurosurgery, University of California San Francisco, Benioff Children’s Hospitals, San Francisco, California, USA; Children’s Cancer Research Laboratory, Center of Genetic Epidemiology, Keck School of Medicine, University of Southern California, Los Angeles, California, USA; Department of Neurosurgery, Keck School of Medicine, University of Southern California, Los Angeles, California, USA; Department of Neurosurgery, Keck School of Medicine, University of Southern California, Los Angeles, California, USA; Brain Tumor Center, University of Southern California, Los Angeles, California, USA; Brain Tumor Center, University of Southern California, Los Angeles, California, USA; Children’s Cancer Research Laboratory, Center of Genetic Epidemiology, Keck School of Medicine, University of Southern California, Los Angeles, California, USA; Department of Biochemistry and Molecular Medicine, Norris Comprehensive Cancer Center, Keck School of Medicine, University of Southern California, Los Angeles, California, USA; Department of Pathology and Laboratory Medicine, Children’s Hospital of Philadelphia Research Institute, Philadelphia, Pennsylvania, USA; Department of Neurosurgery, Johns Hopkins University School of Medicine, Baltimore, Maryland, USA; Department of Biochemistry and Molecular Medicine, Norris Comprehensive Cancer Center, Keck School of Medicine, University of Southern California, Los Angeles, California, USA; Department of Neurosurgery, Keck School of Medicine, University of Southern California, Los Angeles, California, USA; Brain Tumor Center, University of Southern California, Los Angeles, California, USA

**Keywords:** epigenetics, FOXC1, malignant transformation, meningioma

## Abstract

**Background:**

Meningiomas are the most common primary brain tumor. Though typically benign with a low mutational burden, tumors with benign histology may behave aggressively and there are no proven chemotherapies. Although DNA methylation patterns distinguish subgroups of meningiomas and have higher predictive value for tumor behavior than histologic classification, little is known about differences in DNA methylation between meningiomas and surrounding normal dura tissue.

**Methods:**

Whole-exome sequencing and methylation array profiling were performed on 12 dura/meningioma pairs (11 WHO grade I and 1 WHO grade II). Single-nucleotide polymorphism (SNP) genotyping and methylation array profiling were performed on an additional 19 meningiomas (9 WHO grade I, 5 WHO grade II, 4 WHO grade III).

**Results:**

Using multimodal studies of meningioma/dura pairs, we identified 4 distinct DNA methylation patterns. Diffuse DNA hypomethylation of malignant meningiomas readily facilitated their identification from lower-grade tumors by unsupervised clustering. All clusters and 12/12 meningioma-dura pairs exhibited hypomethylation of the gene promoters of a module associated with the craniofacial patterning transcription factor FOXC1 and its upstream lncRNA FOXCUT. Furthermore, we identified an epigenetic continuum of increasing hypermethylation of polycomb repressive complex target promoters with increasing histopathologic grade.

**Conclusion:**

These findings support future investigations of the role of epigenetic dysregulation of FOXC1 and cranial patterning genes in meningioma formation as well as studies of the utility of polycomb inhibitors for the treatment of malignant meningiomas.

**Key Points**

- Diffuse DNA hypomethylation of malignant meningioma differentiates from lower grades by unsupervised clustering.
- All clusters exhibited hypomethylation of gene promoters of the craniofacial patterning transcription factor FOXC1 and its upstream lncRNA FOXCUT.

**Importance of the Study**

Although several studies have focused on changes in DNA methylation between meningioma subtypes, here we examine the changes in DNA methylation between meningiomas and normal dura. Unsupervised clustering identified 4 methylation subtypes with all WHO grade III meningiomas clustering together. DNA hypermethylation affected the promotors of genes regulating cell adhesion and central nervous system differentiation. All meningioma subtypes exhibited hypomethylation affecting the craniofacial patterning transcription factor FOXC1 and its upstream lncRNA FOXCUT. These results provide insight into the patterns of epigenetic dysregulation accompanying meningioma formation and support further investigations into the role of the FOXCUT/FOXC1 complex in tumorigenesis.

Meningiomas are the most common primary central nervous system tumor representing 36.4% of all primary brain tumors.^1^ Although 80% of meningiomas are WHO grade I tumors, the location and invasion patterns of many prevent complete resection leading to high rates of recurrence and progression.^2^ Although it has long been observed that a subset of histologically benign tumors behave aggressively, the 2021 WHO CNS guidelines have included several molecular markers that improve subtyping and prognostication, including SMARCE1, BAP1, KLF4/TRAF7, activating TERT promoter mutations, and CDK2NA/B deletion.^3–8^ There are no targeted drugs with proven efficacy.^9^

Though meningiomas are classically attributed to *NF2* disruptions, sequencing studies have identified mutations in *PI3KCA*, *AKT1*, *SMO*, *KLF4*, *TRAF7*, *POLR2A*, *CDKN2A*, BAP1, and the DREAM complex.^10–16^ Familial meningioma syndromes have been attributed to mutations involving the SWItch/sucrose non-fermentable (SWI/SNF) chromatin remodeling complex components *SMARCB1* and *SMARCE1*.^8,17–21^ Although deletion of chromosome 22q is by far the most common chromosomal copy number alteration identified in meningiomas, multiple groups have identified other recurrent alterations, including deletion of chromosomes 6p, 10p, 14q, 1p32, 1p21, and whole arm deletion of 1p.^22–24^

The low mutational burden observed in meningiomas prompted a search for epigenetic drivers, leading to the observation that DNA methylation profiling outperforms histopathologic classification in prognostication.^17,25^ Despite investigations of DNA methylation patterns between tumor subtypes, to date the epigenetic changes between meningiomas and the surrounding normal tissue have not been investigated.^26^ Utilizing meningioma/dura pairs to control for drift in DNA methylation between patients and tissues of different origin, this multimodal study was undertaken to investigate the mutational burden of coding genes, copy number variants, and epigenetic alterations between individual tumors and the adjacent normal dura that may contribute to tumorigenesis.

## Methods

### Human Subjects and Ethical Considerations

All experimental procedures were approved by our Institutional Review Board, and informed consent was obtained prior to surgery (HS-07-00697, HS-18-00573, HS-20-00261, HS-11-00385, HS-12-00474, HS-11-00520).

### Tissue Collection and DNA Isolation and Bisulfite Conversion

Tumor and dura samples were collected at the time of surgery after the allocation of adequate sample for clinical purposes. StealthStation 7 (Medtronic, Inc.) was used to plan a craniotomy with 2-3 cm of radiographically normal dura around the edges of the tumor. Tissue was classified as normal dura based on visual inspection and neuro-navigation mapping. Normal dura samples were taken from a minimum of 1 cm from the radiographic borders of the mass. Given the small amount of available tissue, the assignment of sample to dura was based on neuro-navigation and gross visual inspection only. DNA was isolated using DNeasy Blood & Tissue Kit (Qiagen). Bisulfite conversion was performed with an EZ DNA Methylation Kit (Zymo Research).

### Statistical Analysis

Statistical analysis was conducted using the base stats package from R version 3.5.3.

### Whole-Exome Sequencing

Sequencing libraries were prepared with the Agilent SureSelect Exome library v4 and sequenced on an Illumina HiSeq2500 in High Output mode (125 bp paired-end). Alignment of FASTQ files was performed based on the Genome Analysis Toolkit (GATK) best practices guidelines.^27^ Briefly, unmapped reads were aligned to Human Reference genome 38 using BWA (19451168). GATK 4.1.0.0 was used to mark duplicates and perform base quality score recalibration. Resulting BAM files were used for somatic variant calling.

### Somatic Variant Calling

Somatic variant calling analysis was performed based on the GATK best practices.^27^ Somatic variants detected in tumor compared to adjacent normal dura were called using Mutect2 followed by calculation of contamination (CalculateContamination function) and filtering of variants (FilterMutectCalls function) in the GATK environment.^28^ Using the Ensembl gene annotations, variants were annotated with Annovar and allele frequency was calculated with exonic gnomAD.^29^ Only exonic variants with a minimal allelic fraction (MAF) <0.0001 in gnomAD, a somatic allelic fraction >0.05, and at least 5 somatic variant supporting alleles were included. Only variants from 73 cancer-associated genes associated with meningioma were considered.^12^

### Copy Number Analysis

For all 31 samples, copy number variations (CNVs) were called from the Illumina 450K methylation using the R Bioconductor package *conumee* for 15 080 bins with a bin size of 50-5000 kb to generate intensity ratios between each meningioma (n = 31) and pooled dura controls (n = 12). Conumee generates ratios using a circular binary segmentation algorithm to call variants from the sum of the methylated and unmethylated signals for each probe.^30^ Raw IDAT files were imported with the R Bioconductor package minfi and the preprocessIllumina normalization function was used to generate a GenomicRatioSet object for input into *conumee*. To generate ratios for meningioma-specific detail lists, the list of oncogenes included with the *conumee* package was appended to include genes previously identified as mutated in meningiomas, components of the BAF (SWI/SNF) complex, and chromosomal regions that have been associated with aggressive behavior in meningiomas.^12,23,24,31,32^ Using a ratio cutoff of ±0.25 for calling copy number variations from the tumor/dura bin ratios generated by *conumee*, a chromosome heatmap was generated using the R package ComplexHeatmap.^33^ Additionally, tumor/dura ratios generated for the loci included in the detail list were used with a cutoff of ±0.25 to call loci as amplified (≥+0.25), neutral (−0.25 to +0.25), or deleted (≤−0.25) using custom scripts written in R.

As a secondary method of CNV-calling for patients 13-31, IDAT files from an Illumina HumanOmni-Express v1.2 BeadChip SNP array were imported into the Illumina GenomeStudio v2.0.5 genotype analysis software for calling of CNVs and regions with loss of heterozygosity (LOH) using the cnvPartition CNV Analysis plugin v3.2.1 with default settings to identify CNVs and regions of LOH based on deviations from the expected B-allele frequency and the log intensity ratios (logR) of tumor vs normal tissue (Illumina, CA). Called CNVs and regions with LOH were output to a bookmark list in text format and then imported into an R GRanges object to determine the overlap of called CNVs with loci of interest. Deleted loci were defined as loci overlapping with a CNV with a copy number <2 and amplified loci were defined as loci overlapping with a CNV with a copy number >2.

### Genome-Wide DNA Methylation Profiling

To identify epigenetic alterations linked to meningioma, DNA methylation for 12 dura and meningioma pairs and an additional 19 meningiomas of varying grades was investigated using the Illumina HM450 methylation arrays (total 12 dura, 31 meningiomas). Prior to methylation analysis, purified DNA was bisulfite converted using the Zymo EZ DNA Methylation Kit according to the manufacturer’s instructions (Zymo, Irvine, CA). Control and bisulfite converted DNA were subsequently analyzed using an Illumina Infinium HumanMethylation450 BeadChip array according to the manufacturer’s instructions. Outputs were analyzed in R (version 3.5.3) using custom scripts developed in the RStudio integrated development environment (RStudio, Boston, MA). All samples were subjected to quality control using the minfi getQC and ChAMP champ.qc functions.^34,35^ Raw IDAT files were uploaded using minfi (version 1.28.4) and normalized using the functional normalization algorithm (preprocessFunnorm) specifically developed for tumor analysis.^34^

Probes were subsequently filtered according to an updated analysis of the HM450 probe mapping to the hg38 genome build using the recommended general masking.^36^ The remaining probes were converted to β-values and M-values using the minfi getBeta and getM functions, respectively. Based on inspection of the density distribution plot of β-values of all samples, probes with β ≥ 0.3 were considered methylated or partially methylated and β < 0.3 were considered unmethylated (Supplementary Figure 2a).

### Principle Component Analysis and Singular Value Decomposition of Phenotypic Datasets

To determine the variability between samples attributable to each principal component, the magnitude of the principal components was determined using the base R function prcom and a scree plot was generated in the R package factoextra (version 1.0.6) using the function fviz_eig. To determine the respective contributions of sample characteristics, including batch effects and patient phenotypic variables, to the variability of β-values between samples, a singular value decomposition (SVD) was performed using the champ.sva function from the R package ChAMP.^37^

### Probe-Wise Differential DNA Methylation Analysis

Prior to analysis, β-values were filtered to remove probes with similar values across samples. Probes with mean β-value <0.2 and at least 10% of tumor β-value >0.2 and probes with mean β-value >0.8 and at least 10% of tumor β-value <0.7 were included for analysis. Empirical Bayes testing was used to conduct probe-wise differential methylation analysis using M-values of filtered probes for tumor samples (n = 31) vs normal dura samples (n = 12) using the lmFit and eBayes functions from the linear models for microarray data (*limma*) package in R (version 3.38.3).^38^ A false discovery rate cutoff (Benjamini-Hochberg correction) of 0.05 was used to identify probes as differentially methylated probes (DMPs) between tumor and normal.^39^

### Unsupervised Clustering of DNA Methylation Datasets

Euclidean distance matrices for tumor samples were calculated using DMPs using the get_dist function in the R package factoextra (version 1.0.6).

Unsupervised k-means clustering was performed on the resulting distance matrices using the R package ConsensusClusterPlus with a maxK of 20 and 1000 reps (version 1.46.0).^40^

### DNA Methylation Probe Mapping

To identify global changes in methylation for each methylation cluster, hypermethylated probes were defined as β _tumor_-mean(β _dura_) ≥ 0.2 and hypomethylated probes as β _tumor_-mean(β _dura_) ≤ –0.2. To test for differences in the number of hyper- and hypomethylated probes per tumor in each methylation cluster, an analysis of variance (ANOVA) was performed with Tukey’s post hoc comparison with a significance threshold of *P* < .05.

### Promoter-Wise Differential DNA Methylation Analysis

A custom R script was generated for the analysis of promoter methylation. Based on the analysis of the probe density plot, probes for each sample were called methylated if the β-value was ≥0.3 and unmethylated if a probe β-value was <0.3. Using the hg38 mapping of probes described by Zhou et al, probes were subsequently mapped to the gene promoters of the GENCODE v29 primary assembly defined at ±2 kb from the transcription start site yielding a total of 32 355 genes whose promoters overlapped with at least 1 probe. The percentage methylation of the promoters in each sample was calculated as ( #  methylated probes × 100)/( #  overlapping    probes).

For the identification of differentially methylated promoters between meningiomas (n = 31) and dura (n = 12), a contrast model was built contrasting meningiomas to dura (n = 12 dura samples). Differentially methylated promoters between each cluster and dura were identified with a contrast model between each cluster and dura. Differentially methylated promoters were identified using the lmFit, eBayes, and decideTests functions in *limma* with a false discovery rate cutoff of <0.05 using the Benjamini-Hochberg correction.^41^

Meningioma/dura pairs were utilized for the analysis of methylation changes between *NF2* altered and wildtype tumors. CNV and exome-seq analysis identified 5 meningiomas with *NF2* deletions or mutations and 7 *NF2* wildtype meningiomas. A contrast model was built for each group in *limma* to analyze for differential promoter methylation between tumors of each subtype and normal dura from the same patient, accounting for the paired nature of the samples in the model. The dura sample from patient 4 clustered with dura samples but exhibited a more diffuse hypermethylation of probes relative to other samples (Figure 2a). Examination of the postoperative MRI showed abnormal thickening of the dura that extended well beyond the edges of the craniotomy. Given concern for contamination of the dura sample with tumor cells, this sample was excluded from the paired analysis. The meningioma sample was included for direct comparison of promoter methylation between *NF2* altered and *NF2* wildtype meningiomas.

**Figure 1.**
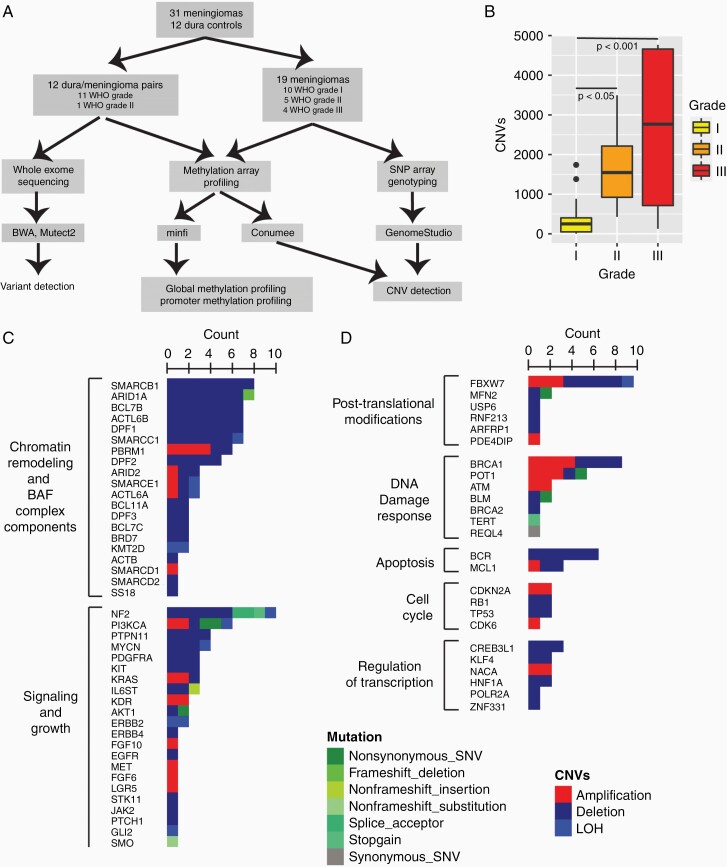
Meningiomas exhibit a low mutational burden. Whole-exome sequencing (WES) of 12 meningiomas identified 0-3 mutations per tumor. (a) Lollipop plot of *NF2* mutations. (b) Lollipop plot of identified PIK3CA mutation including a glutamic acid (E) to lysine (K) mutation at amino acid 542 and a histidine (H) to alanine (A) at amino acid 1047. (c) Cumulative alterations identified in common oncogenes and components of the SWItch/sucrose non-fermentable pathway (SWI/SNF) as identified via WES (12/31 meningiomas), copy number variant (CNV) analysis using *conumee* (31/31 meningiomas), and single-nucleotide polymorphism genotyping (19/31 meningiomas). Red: amplification, Blue: deletion, Green: mutation, Light green: Loss of heterozygosity (LOH). (d) Count of CNVs in each tumor grade. ANOVA with Tukey’s post hoc analysis, *P* < .05 for significance. FERM—4.1 protein, ERM: Ezrin-Radixin-Moesin, P85B: p85-binding domain, RBD: RAS-binding domain, C2: C2 putative membrane-binding domain, PI4kinase: phosphoinositide 4-kinase domain.

### Molecular Signatures Database Analysis of Gene Sets

The curated (C2, including KEGG pathway and Reactome) and C5 (Gene Ontology [GO]) gene sets were downloaded using the msidgbr package in R (version 7.2.1) for gene set enrichment analysis.^42,43^ Ranked lists of significantly hypermethylated or hypomethylated gene promoters were analyzed using the enricher function in the R package clusterProfiler version 3.10.1, and results were plotted using the R package enrichplot version 1.2.0.^44^

### Cancer Bioportal Analysis

To identify whether differentially methylated genes play a role in other cancers, a set of 15 highly differentially methylated genes were submitted as a query to cBioPortal using a curated set of 184 non-redundant studies to generate a custom OncoPrint and cancer types summary.^45,46^

### RNA Extraction and qPCR

Total RNA was extracted from normal dura tissue and 10^6^ cells from a cultured meningioma cell line generated from a 47-year-old patient with a WHO grade II meningioma using the RNeasy Plus Mini Kit (Qiagen). A normalized 1 µg of RNA was used as a template for first-strand cDNA synthesis with the iScript Select cDNA Synthesis Kit (Bio-Rad) with the kit-provided random primer per kit directions. FOXC1 primers were obtained from Thermo Fisher Scientific/Applied Biosystems (“Rack ID P03329039”) and 18s primer sequence were from published reference genes.^47^ qPCR was run on a Roche LightCycler 96, using a SYBR Green filter, 1 cycle of 95°C for 600 seconds, 45 amplification cycles with steps of 95°C for 10 seconds, 60°C for 10 seconds, 72°C for 10 seconds, 1 primer melting cycle of 95°C for 10 seconds, 60°C for 60 seconds, 97°C for 1 second with acquisition every 5°C. Results were analyzed with LightCycler 96 Software using the mean 2^−(∆∆Ct)^ method.

### Culture of Meningioma Cell Lines

Samples were received fresh from the operating room immersed in Advanced DMEM/F12 (Thermo Fisher Scientific, Cat. # 12634010) and immediately prepared for processing. Tumor tissues were first processed by finely mincing the sample followed by chemical digestion in 0.25% Trypsin-EDTA (Thermo Fisher Scientific, Cat. # 25200056) for 2-3 minutes. Enzymatic digestion was halted using FBS-supplemented medium and the tissue mixture was centrifuged at 1000 rpm for 4 minutes prior to in collagen-coated tissue culture flasks, as previously described.^48^ Cells were cultured in 50% glutamine-supplemented medium (Advanced DMEM/F12, 10% fetal bovine serum (Omega Scientific, Cat. # FB-02), 1x Glutamax (Thermo Fisher Scientific, Cat. # 35050061), 1x Antibiotic-Antimycotic (Life Technologies, Cat. # 15240062)), and 50% Neurobasal-A medium ((Neurobasal-A (Life Technologies, Cat. # 10888022), 1x Glutamax, 1x B-27 Supplement (Thermo Fisher Scientific, Cat. # 17504044), 1x Antibiotic-Antimycotic) supplemented with 10 µM Y-27632 dihydrochloride (Tocris, Cat. # 1254) every 2 days.

### Histology and Image Analysis

About 5 µm paraffin-embedded sections were deparaffined with Histo-Clear II (National Diagnostics) and rehydrated with graded alcohol solutions ranging from 100% to 70%. Antigen retrieval was performed by exposing deparaffined samples to Tris-EDTA, pH 9.0 (Genemed), and boiling in a microwave for five 2-minute incubations. Tissue sections were rinsed in Tris Buffered Saline (TBS) then permeabilized with 0.1% Triton X-100 in TBS then blocked with 1% bovine serum albumin in TBS-Tween-20 (TBST). Rabbit recombinant monoclonal FOXC1 antibody (Abcam, ab227977) was diluted in the blocking buffer (1:500) and incubated overnight. After a wash in TBS, anti-rabbit, Alexa-647-conjugated secondary antibody (Thermo Fisher Scientific) was diluted in blocking buffer (1:1000) and incubated on the samples for 1.5 hours. Samples were washed in TBS and then mounted with Prolong anti-fade with DAPI (Thermo Fisher Scientific) and a coverslip. Fluorescence was visualized using a BZ-9000 microscope (Keyence). Image analysis was performed using NIH ImageJ software version 1.53k and Java version 1.8.0_176 (64-bit) using the *Image* > *Adjust* > *ColorThreshold* and *Analyze Particles* functions using a particle size of 0.05-0.4 to identify the fraction of cells expressing FOXC1. ANOVA and Tukey’s post hoc analysis utilized the R base stats package.

## Results

### Patient Characteristics

Our cohort included 31 surgically resected meningiomas (21 grade I, 6 grade II, and 4 grade III) (Supplementary Tables 1 and 2). Our cohort included 31 meningiomas (21 WHO grade I (68%), 6 WHO grade II (20%), and 4 WHO grade III (13%)) with a mean age of 52.2 years (Figure 1a, Supplementary Table 1). None of the patients carried a diagnosis of neurofibromatosis. There was a female sex predominance with 25 (80.6%) females and 6 males (19.4%). A chi-squared test of interdependence showed that males were significantly more likely to have higher-grade tumors (*X*^2^(1, N = 31) = 4.03, *P* = .045). Clinical data were available for 15/31 patients (Supplementary Table 2) with a mean follow-up of 3.8 ± 2.8 years (median 3 years). Of these, 4/15 samples were obtained from recurrent meningiomas. Of the 15 patients for whom clinical data were available, 6 patients (40%) were either undergoing a repeat surgery for recurrent tumor or developed progression of their tumor within the available follow-up period.

### Recurrent Genetic Alterations

Mean coverage exceeded 110 for all samples. An average of >91% of targets per sample had at least 80× coverage (Supplementary Tables 3 and 4). Whole-exome sequencing of 12 tumor and dura pairs (11 grade I and 1 grade II) identified 0-3 mutations per meningioma (Supplementary Table 5). Mutations in *NF2* were the most frequently observed (n = 3, 25%) including one nonsense mutation (Q470*) and splice acceptor mutations in exons 3 and 5 (Figure 1c, Supplementary Figure 1a). Two patients had *PI3KCA* mutations including an E542K substitution in the accessory domain and a C-terminal histidine to arginine substitution (H1047A), both loci associated with activating mutations (Figure 1c, Supplementary Figure 1b). Mutations clustered in two groups, those affecting the PI3KCA/AKT1 pathways (*NF2*, *AKT1*, *PI3KCA*) and those affecting DNA maintenance and repair machinery (telomerase [*TERT*], DNA helicase Bloom syndrome protein [*BLM*], protection of telomeres [*POT1*]) (Supplementary Table 3). Other identified mutations involved a diverse set of signaling pathways including the SWI/SNF chromatin remodeling complex (*ARID1A*), the Hedgehog pathway (*SMO*), the WNT/β-catenin pathway (*AMER1*), and the IL6/JAK/STAT3 pathway (*IL6ST*) (Figure 1c).

### Progressive Genomic Instability

Grade I meningiomas had significantly fewer bins with copy number variants (CNVs) than grade II or III meningiomas (I: 372 ± 467, II: 1694 ± 1121, III: 2607 ± 2435, ANOVA *P* < 10, Tukey’s post hoc analysis: I vs II: *P* = .02, I vs III: *P* < 10, II vs III: *P* = .35, Figure 1d, Supplementary Figure 1d). Deletion and LOH events were found frequently at chromosome 1p (15/31, 48%) and chromosome 22q (10/31, 32%) with co-alterations of 1p/22q in 4/21 (19%) grade I meningiomas and 6/10 (60%) grade II/III meningiomas (Supplementary Figure 1e). Alterations affecting the SWI/SNF chromatin remodeling complex were identified in 18/31 (58%) meningiomas and affected 8/10 grade II/III meningiomas (Supplementary Figure 1f).

### Identification of Methylation Clusters

**Figure 2.**
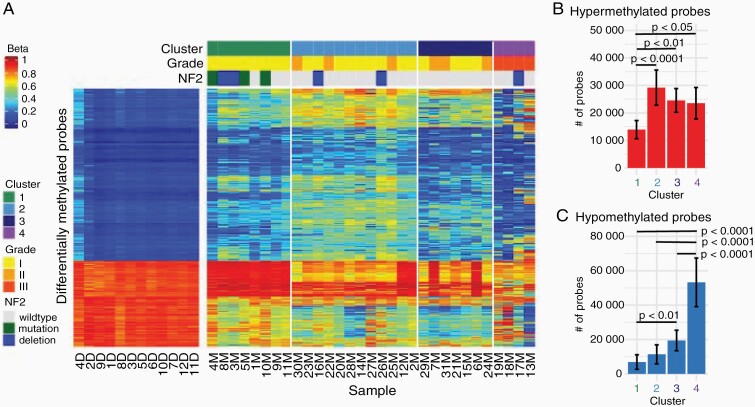
Unsupervised clustering identifies methylation subgroups. (a) K-means clustering of differentially methylated probes identifies 4 methylation clusters. (b) Mean count of hypermethylated probes per meningioma in each methylation cluster. (c) Mean count of hypomethylated probes per meningioma in each methylation cluster. Error bars: standard deviation.

Of the 421 368 probes that passed filtering criteria, 1607 DMPs were identified of which 80% (1280) were hypermethylated and 20% (327) were hypomethylated (Figure 2a, Supplementary Figure 2a).^36^ K-means clustering identified k = 4 methylation clusters (Figure 2a, Supplementary Figure 2b and c). Tumor grade was significantly associated with DNA methylation-based clusters (Supplementary Figure 2d). Cluster 1 was enriched for grade I tumors and contained 5/7 grade I tumors with *NF2* alterations while cluster 4 contained all grade III tumors (Pearson’s chi-squared test, χ ^2^ = 35.666, df = 6, *P* = 3.2 × 10^−6^).

Principle component analysis indicated that 94.4% of the variability in DNA methylation between samples could be attributed to the first principal component (Supplementary Figure 2e). The primary drivers of the first and second principal components as identified by SVD were cluster (*P* < 10^−10^) and grade (*P* < 10^−10^) followed by phenotype (*P* < 10), Supplementary Figure 2e). A history of meningioma recurrence/progression and deletions of chromosome 1p (chr1p/chr1p36 alterations or ARID1A alterations, *P* < 10) were significant drivers of the second principal component. *NF2* status was a weak driver of the third principal component (*P* < .05).

The number of hypermethylated and hypomethylated probes relative to dura differed significantly between clusters (ANOVA with Tukey’s post hoc analysis, Figure 2b and c, Supplementary Tables 3 and 4). Cluster 1 meningiomas had significantly fewer hypermethylated probes than clusters 2-4. Clusters 4 meningiomas had significantly more hypomethylated probes than clusters 2-4 and cluster 3 exhibited significantly more hypomethylated probes than cluster 1.

### Epigenetic Dysregulation of Promoters

**Figure 3.**
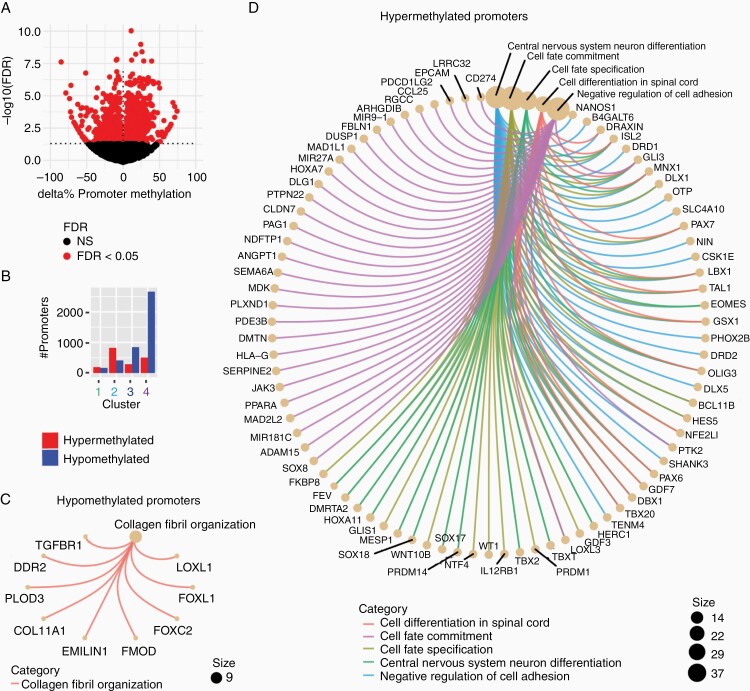
Meningiomas exhibit alterations in DNA methylation of promoters. (a) Changes in promoter methylation between meningiomas and normal dura. *x*-axis: Δ% methylation of gene promoters in meningiomas vs dura. *y*-axis: −log_10_ FDR (Benjamini-Hochberg). (b) Count of differentially methylated promoters in each cluster relative to normal dura. Red: hypermethylated. Blue: hypomethylated. (c) Hypomethethylated genes were enriched for GO:0030199 collagen fibril organization (*P* < .05, *q*-value < 0.2, BH). (d) MSigDB Gene Ontology gene sets were enriched for hypermethylated promoters (*P* < .05, *q*-value < 0.2, BH).

Probes were mapped to ±2 kb of GENCODE v29 gene transcription start sites to calculate the percentage of methylated probes in each promoter. Significantly differentially methylated promoters were identified in *limma* using a false discovery rate of 5% which identified 1314 hypermethylated and 776 hypomethylated promoters (Figure 3a).^41^ Patterns of differential promoter methylation in each cluster were similar to patterns of differential probe methylation between clusters (Figure 3b).

Hypomethylated promoters were only enriched for the GO term collagen fibril organization which contains the craniofacial patterning transcription factors *FOXC1* (Figure 3c). Among the most significantly hypomethylated gene promoters is *FOXCUT*, a lincRNA transcribed (Supplementary Table 6) from the upstream promoter region of the forebrain patterning gene *FOXC1*. *BMP4* and *PITX2*, interaction partners of the *FOXC1* craniofacial patterning network, were also hypomethylated (Supplementary Figure 3a–c).

Hypermethylated promoters were significantly enriched for Gene Ontology terms involved in central nervous system patterning, cell fate specification, and negative regulation of cell adhesion including particularly networks of developmental transcription factors, such as *PAX6*, *TBXT/Brachyury*, and the *Hox* clusters (Figure 3d).

Clusters 3 and 4 exhibited hypomethylation affecting gene promoters associated with blood vessel development and G-protein-coupled receptor signaling, respectively (Supplementary Figure 3d). Cluster 4 tumors were readily identified by diffuse hypomethylation across the genome. Although there were no enriched GO terms among the hypermethylated promoters of clusters 1-3, there was a continuum of hypermethylation of the target promoters of the polycomb repressive complex 2 (PRC2) between cluster 2, consisting of grade I/II tumors, and cluster 4 which contained all grade III tumors (Supplementary Figure 3e). All clusters exhibited hypermethylation of classical PRC2 targets such as the HOXC cluster and TBXT/Brachyury (Supplementary Figure 3f and g). Of note, this pronounced focal hypermethylation of PRC2 targets in cluster 4 meningiomas was observed despite diffuse hypomethylation.

Forty genes were hypomethylated relative to dura in all methylation clusters, including 18 protein-coding genes and 5 lincRNAs. Twenty-eight genes were hypermethylated in all clusters, including 12 protein-coding genes and 5 long intergenic non-coding RNAs (lincRNAs). As many identified genes were poorly represented in the literature, a list of 16 genes with differentially methylated promoters in all clusters was submitted as a query to the cBioPortal cancer genomics portal using a sample of 184 non-redundant cancer genomics studies. A total of 5560 (12%) of the combined 48 081 samples from 45 604 patients had an alteration in at least one of the submitted genes (Supplementary Figure 3). At least one of the submitted genes was altered in >30% of ovarian epithelial tumors, bladder tumors, sarcomas, endometrial carcinomas, non-melanoma skin cancers, and melanomas (Supplementary Figure 3h and i).

### Methylation Patterns Related to *NF2* Alterations

**Figure 4.**
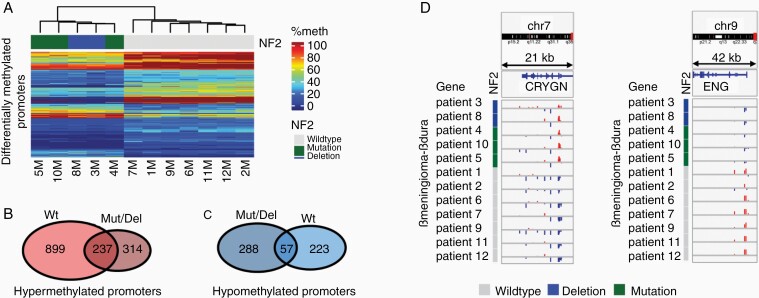
*NF2* altered and wildtype tumors exhibit differential DNA methylation of promoters. (a) Heatmap of percentage methylation of 423 differentially methylated promoters in *NF2* altered vs wildtype tumors. Legend: Percentage of methylated probes overlapping each promoter. (b, c) Venn diagram of hypermethylated (b) and hypomethylated (c) promoters in *NF2* wildtype (Wt, n = 7 patients) and *NF2* altered (Mut/Del, n = 4 patients) meningiomas relative to normal dura from the same patient. (d) β-value_meningioma_−β-value_dura_ for probes mapping the promoter region of the *endoligin* (*ENG*) and *crystallin gamma N* (*CRYGN*) genes for meningioma/dura pairs from 12 patients (5 *NF2* altered, 7 wildtype).

Exome sequencing and copy number analysis of our meningioma/dura pairs identified 5 tumors with *NF2* alterations (3 mutations and 2 deletions) and 7 *NF2* wildtype meningiomas. An analysis of promoter methylation in *NF2* altered vs wildtype yielded 98 gene promoters that were hypermethylated in *NF2* altered meningiomas relative to wildtype tumors and 325 gene promoters that were hypomethylated in *NF2* wildtype tumors (Figure 4a; Supplementary Figure 4). Compared to adjacent normal dura from the same patient, *NF2* wildtype tumors were found to have 1136 hypermethylated and 345 hypomethylated promoters while *NF2* altered tumors were found to have 551 hypermethylated and 280 hypomethylated promoters (Figure 4b and c). Two hundred thirty-seven gene promoters were hypermethylated relative to dura regardless of *NF2* status and 57 promoters were hypomethylated regardless of *NF2* status, including FOXCUT and BMP4.

Hypermethylated promoters in *NF2* altered tumors were enriched for GO gene sets associated with hypertrophic changes of the skin and nails (Figure 4b). Hypomethylated GO gene sets were enriched for genes involved in the patterning of blood vessels and limb abnormalities such as the endoglin gene *ENG/CD105*, a mesenchymal stem cell marker that acts as an accessory protein in the TGF-β superfamily (Figure 4c and d).^49^

### Increased Expression of FOXC1 in Meningiomas Relative to Dura

**Figure 5.**
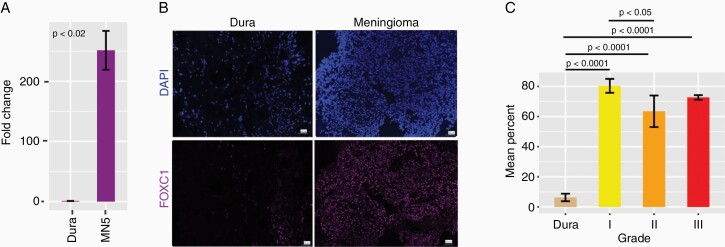
Increased FOXC1 expression in meningiomas. (a) qPCR shows significantly greater FOXC1 mRNA expression in a primary meningioma cell line than dura. (b) ×20 magnification of FOXC1 expression in primary meningioma vs dura from the same patient. (c) Quantification of the mean percentage of nuclei expression FOXC1 in meningiomas (grade I: n = 4, grade II: n = 3, grade III: n = 3) vs dura (n = 3) shows significantly higher expression relative to dura in all tumor grades. Mean percentage ± standard error of the mean. Length of the scale bar represents 50 µm.

There was a 251-fold higher (*P* = .01) expression of FOXC1 mRNA in a primary meningioma cell line from a 47-year-old patient with a grade III meningioma relative to dura from the same patient (Figure 5). Staining of dura from 4 patients and 9 meningiomas (4 grade I, 3 grade II, 2 grade III) showed a statistically significant difference in the percentage of cells expressing FOXC1 (Figures 5 and 6, dura vs I/II/III *P* < .001, I vs II *P* < .05, I vs II/III NS).

## Discussion

The dura and meninges play significant roles in the development of the cranial vault and cerebral cortex, yet dura remains an understudied tissue, and patterns of epigenetic dysregulation in meningiomas relative to surrounding normal tissue have not been described.^50–52^ These studies provide insight into the subtypes of epigenetic dysregulation in meningiomas as well as the changes between tumor and dura that are common to all tumors.

Genetic alterations affecting the SWI/SNF chromatin remodeling complex were highly represented, particularly in grade II/III tumors. This may be a bystander effect of the genetic instability in higher-grade tumors, however, the SWI/SNF complex is believed to oppose the actions of the PRC2 complex and SWI/SNF complex components and mutations of the SWI/SNF complex components SMARCB1/SMARCE1 mutations are associated with syndromes of multiple meningiomas.^8,17,18^ Biallelic deletions of SMARCB1 are the causative mutation of atypical teratoid rhabdoid tumor, an aggressive pediatric tumor that exhibits a similar phenotype to meningiomas with few mutations and pronounced hypermethylation of PRC2 targets.^53^ Hypermethylation of PRC2 target promoters was observed in multiple methylation clusters and was particularly prominent in malignant meningiomas despite diffuse DNA hypomethylation across the remainder of the genome. The role of the SWI/SNF complex in meningioma formation warrants further investigation to determine whether some subtypes of these tumors may be responsive to PRC2 inhibitors.

These studies provide insight into the patterns of epigenetic dysregulation in meningiomas and characterized epigenetic changes that are common across methylation clusters. In particular, all tumor subtypes exhibited hypomethylation affecting the promoter of the transcription factor FOXC1 and its upstream lncRNA transcript FOXCUT. The DNA hypomethylation of this module is accompanied by a significantly higher expression of the FOXC1 mRNA and protein in tumors compared to dura suggesting. Mutations of FOXC1 in humans lead to severe craniofacial abnormalities and FOXC1-mutant mice exhibit severe cranial abnormalities including the absence of forebrain dura.^54^ Additionally, FOXCUT and FOXC1 have been shown to form mRNA-lncRNA complex that leads to upregulation of the FOXC1/PI3K/AKT pathway and play a role in a diverse set of cancers. These findings warrant future studies to determine whether FOXCUT/FOXC1 drive transcriptional module in meningiomas.^55^

## Supplementary Material

vdac084_suppl_Supplementary_Table_S1

vdac084_suppl_Supplementary_Table_S2

vdac084_suppl_Supplementary_Table_S3

vdac084_suppl_Supplementary_Table_S4

vdac084_suppl_Supplementary_Material
