# Corrigendum to: Epigenetic dysregulation in meningiomas

**DOI:** 10.1093/noajnl/vdac190

**Published:** 2023-01-06

This is a corrigendum to: Michelle A Wedemeyer, Ivo Muskens, Ben A Strickland, Oscar Aurelio, Vahan Martirosian, Joseph L Wiemels, Daniel J Weisenberger, Kai Wang, Debraj Mukerjee, Suhn K Rhie, Gabriel Zada, Epigenetic dysregulation in meningiomas, *Neuro-Oncology Advances*, Volume 4, Issue 1, January-December 2022, vdac084, https://doi.org/10.1093/noajnl/vdac084

In the originally published version of this manuscript Gabriel Zada was listed as the corresponding author in error. Both Gabriel Zada and Suhn K. Rhie should have been listed as co-corresponding authors.

This error has been corrected.

