# Supplementary material for: Epigenetic dysregulation in meningiomas
Source: Neurooncol Adv. 2022 Jun 6;4(1):vdac084. doi: 10.1093/noajnl/vdac084 (PMC9234763; doi:10.1093/noajnl/vdac084)

Supplemental Information Titles and Legends

**Table 1: Patient Characteristics**

| Patient | Age | Sex | Race | Grade | Location |
| --- | --- | --- | --- | --- | --- |
| 1 | 48 | F | African | I | Sella |
| 2^a^ | 55 | F | Caucasian | I | Sella |
| 3 | 60 | F | Asian | I | Tentorium |
| 4* | 60 | F | Caucasian | I | Parafalcine |
| 5 | 34 | F | Caucasian | I | Convexity |
| 6^b^ | 56 | F | Caucasian | I | Sella |
| 7 | 65 | F | Hispanic | II | Tentorium |
| 8 | 50 | F | Asian | I | Tentorium |
| 9 | 85 | F | Asian | I | Sella |
| 10 | 60 | F | Caucasian | I | Parafalcine |
| 11 | 56 | F | Asian | I | Olfactory Groove |
| 12 | 72 | F | Hispanic | I | Planum Sphenoidale |
| 13 | 58 | M | Caucasian | III | Occipital |
| 14 | 63 | F | Caucasian | I | Temporal |
| 15 | 50 | F | Hispanic | I | Temporal |
| 16 | 61 | F | Hispanic | I | Frontal |
| 17 | 56 | M | Hispanic | III | Frontal |
| 18 | 47 | F | Caucasian | III | Parietal |
| 19 | 32 | F | Asian | III | Olfactory Groove |
| 20 | 35 | M | Hispanic | I | Petrous |
| 21 | 50 | F | Asian | I | Parietal |
| 22 | 55 | M | Hispanic | II | Frontal |
| 23 | 51 | F | Hispanic | I | Clinoid |
| 24 | 24 | M | Caucasian | II | Frontal |
| 25 | 68 | F | Asian | II | Frontal |
| 26 | 53 | F | Hispanic | I | Sphenoid Wing |
| 27 | 57 | F | Caucasian | I | Temporal |
| 28 | 48 | F | Caucasian | I | Frontal |
| 29 | 60 | F | Hispanic | I | Sphenoid Wing |
| 30 | 44 | F | Hispanic | II | Olfactory Groove |
| 31 | 68 | F | Hispanic | II | Frontal/Temporal |

^a^ Radiographic recurrence detected

^b^ Underwent reoperation

**Table 2: Clinical Characteristics**

| **Patient** | **EOR** | **Re-operation** | **Recurrence/**  **Progression** | **XRT^a^** | **Chemotherapy** | **Follow-Up (years)** |
| --- | --- | --- | --- | --- | --- | --- |
| 1 | STR | No | No | Adjuvant | No | 3 |
| 2 | STR | Yes | No | Adjuvant | No | 7 |
| 3 | GTR | No | No | No | Yes^b^ | 1 |
| 4 | GTR | Yes | No | No | No | 1 |
| 5 | GTR | No | No | No | No | 5 |
| 6 | STR | Yes | Yes | GKRS | No | 3 |
| 7 | GTR | Yes | No | No | No | 1 |
| 8 | GTR | No | No | No | No | 4 |
| 9 | GTR | No | No | No | No | 1 |
| 10 | GTR | No | No | No | No | 5 |
| 11 | GTR | No | No | No | No | 3 |
| 12 | - | - | - | - | - | - |
| 13 | - | - | - | - | - | - |
| 14 | GTR | No | No | No | No | 4 |
| 15 | - | - | - | - | - | - |
| 16 | STR | Yes | Yes | No | No | 9 |
| 17 | - | - | - | - | - | - |
| 18 | STR | No | Yes | IMRT | No | 9 |
| 19 | GTR | No | No | No | No | 5 |
| 20 | STR | Yes | Yes | GKRS | No | 2 |
| 21 | - | - | - | - | - | - |
| 22 | - | - | - | - | - | - |
| 23 | GTR | No | No | No | No | 2 |
| 24 | GTR | No | No | No | No | 1 |
| 25 | GTR | No | No | No | No | 2 |
| 26 | - | - | - | - | - | - |
| 27 | - | - | - | - | - | - |
| 28 | STR | No | Yes | No | No | 1 |
| 29 | GTR | No | No | No | No | 5 |
| 30 | - | - | - | - | - | - |
| 31 | - | - | - | - | - | - |

^a^ XRT: Adjuvant: post-operatively, GKRS: gamma knife radiosurgery, IMRT: intensity modulated radiation therapy

^b^ Patient 3 underwent treatment with traztuzumab, docetaxel, and pertuzumab for ER/PR- Her2+ breast cancer.

**Table 3: Mean Coverage of WES samples**

| **Sample ID** | **Regions Number** | **Total Region Length** | **Mean Coverage** |
| --- | --- | --- | --- |
| 1D | 185636 | 51189318 | 142.8 |
| 1T | 185636 | 51189318 | 128.2 |
| 2D | 185636 | 51189318 | 127.3 |
| 2T | 185636 | 51189318 | 135.5 |
| 3D | 185636 | 51189318 | 130.6 |
| 3T | 185636 | 51189318 | 136.9 |
| 4D | 185636 | 51189318 | 134.0 |
| 4T | 185636 | 51189318 | 136.7 |
| 5D | 185636 | 51189318 | 137.1 |
| 5T | 185636 | 51189318 | 136.1 |
| 6D | 185636 | 51189318 | 142.3 |
| 6T | 185636 | 51189318 | 805.9 |
| 7D | 185636 | 51189318 | 148.5 |
| 7T | 185636 | 51189318 | 127.3 |
| 8D | 185636 | 51189318 | 139.1 |
| 8T | 185636 | 51189318 | 126.8 |
| 9D | 185636 | 51189318 | 122.0 |
| 9T | 185636 | 51189318 | 141.1 |
| 10D | 185636 | 51189318 | 133.2 |
| 10T | 185636 | 51189318 | 121.6 |
| 11D | 185636 | 51189318 | 129.0 |
| 11T | 185636 | 51189318 | 797.1 |
| 12D | 185636 | 51189318 | 127.9 |
| 12T | 185636 | 51189318 | 135.2 |

**Table 4: Coverage Depth of WES samples**

| **Sample ID** | **100x** | **90x** | **80x** | **70x** | **60x** | **50x** | **40x** | **30x** | **20x** | **10x** | **1x** |
| --- | --- | --- | --- | --- | --- | --- | --- | --- | --- | --- | --- |
| 1D | 67 % | 82% | 89% | 91% | 93% | 94% | 94% | 95% | 95% | 95% | 100% |
| 1T | 70% | 84% | 89% | 92% | 93% | 94% | 95% | 95% | 95% | 95% | 100% |
| 2D | 70% | 85% | 90% | 93% | 94% | 95% | 95% | 95% | 96% | 96% | 100% |
| 2T | 76% | 88% | 92% | 93% | 94% | 95% | 95% | 96% | 96% | 96% | 100% |
| 3D | 73% | 86% | 91% | 93% | 94% | 95% | 95% | 95% | 96% | 96% | 100% |
| 3T | 78% | 89% | 92% | 94% | 95% | 95% | 96% | 96% | 96% | 96% | 100% |
| 4D | 74% | 86% | 91% | 93% | 94% | 94% | 95% | 95% | 95% | 95% | 100% |
| 4T | 75% | 87% | 91% | 93% | 94% | 95% | 95% | 96% | 96% | 96% | 100% |
| 5D | 78% | 89% | 92% | 94% | 95% | 95% | 96% | 96% | 96% | 96% | 100% |
| 5T | 76% | 88% | 92% | 93% | 94% | 95% | 95% | 96% | 96% | 96% | 100% |
| 6D | 77% | 88% | 92% | 94% | 95% | 96% | 96% | 96% | 96% | 96% | 100% |
| 6T | 97% | 98% | 99% | 99% | 99% | 99% | 99% | 99% | 99% | 99% | 100% |
| 7D | 79% | 89% | 93% | 94% | 95% | 96% | 96% | 96% | 96% | 96% | 100% |
| 7T | 71% | 84% | 89% | 92% | 93% | 94% | 94% | 95% | 95% | 95% | 100% |
| 8D | 79% | 89% | 92% | 94% | 95% | 95% | 96% | 96% | 96% | 96% | 100% |
| 8T | 73% | 86% | 90% | 92% | 94% | 94% | 95% | 95% | 95% | 96% | 100% |
| 9D | 66% | 82% | 88% | 91% | 92% | 93% | 94% | 94% | 94% | 95% | 100% |
| 9T | 75% | 87% | 91% | 93% | 94% | 95% | 96% | 96% | 96% | 96% | 100% |
| 10D | 77% | 88% | 92% | 94% | 95% | 95% | 96% | 96% | 96% | 96% | 100% |
| 10T | 72% | 86% | 90% | 92% | 94% | 94% | 95% | 95% | 95% | 96% | 100% |
| 11D | 74% | 87% | 91% | 93% | 94% | 95% | 95% | 96% | 96% | 96% | 100% |
| 11T | 97% | 99% | 99% | 99% | 99% | 99% | 99% | 99% | 99% | 99% | 100% |
| 12D | 74% | 87% | 91% | 93% | 94% | 95% | 95% | 96% | 96% | 96% | 100% |
| 12T | 75% | 87% | 91% | 93% | 94% | 94% | 95% | 95% | 95% | 96% | 100% |

**Table 5: Mutations identified by whole exome sequencing (WES)**

| **Gene** | **ID** | **Genomic** | **Protein** | **RefGene** | **OncoKB** | | | |
| --- | --- | --- | --- | --- | --- | --- | --- | --- |
|  |  |  |  |  | **Mutation Effect** | **Level** | **Tumor** | **Drug** |
| **NF2^a^** | 4 | chr22: C29674903T | Q470* | stopgain | Likely Oncogenic,  Likely Loss-of-function | - | - | - |
|  | 5 | chr22: G29642201- | Splice acceptor | splice acceptor | Likely Oncogenic,  Likely Loss-of-function | - | - | - |
|  | 10 | chr22: G29654656A | Splice acceptor | splice acceptor | Likely Oncogenic,  Likely Loss-of-function | - | - | - |
| **PIK3CA^a,c^** | 6 | chr3: G179218294A | E542K | nonsynonymous SNV | Oncogenic  Gain of Function | 1 | breast | Alpelisib + Fulvestrant |
|  | 1 | chr3: A179234297G | H1047A | nonsynonymous SNV | Oncogenic  Gain of Function | 1 | breast | Alpelisib + Fulvestrant |
| **AKT1^a,d^** | 2 | chr14: C104780214T | E17F | nonsynonymous SNV | Oncogenic  Gain of Function | 3A | breast, ovarian, endometrial | AZD5363 |
| **AMER1** | 10 | chrX: G64191275T | S671* | stopgain | Likely Oncogenic,  Likely Loss-of-function | - | - | - |
| **ARID1A^a^** | 10 | chr1: C26772531- | Q1148Sfs | frameshift deletion | Unknown | - | - | - |
| **BLM** | 1 | chr15: A90798313C | N1112H | nonsynonymous SNV | Unknown | - | - | - |
| **IL6ST** | 12 | chr5: C55947566CTAT | I621II | non-frameshift insertion | Unknown | - | - | - |
| **MFN2** | 2 | chr1: A11998870C | M234L | nonsynonymous SNV | Unknown | - | - | - |
| **POT1** | 4 | chr7: T124863630C | Y89T | nonsynonymous SNV | Likely Oncogenic,  Likely Loss-of-function for Y89C | - | - | - |
| **RECQL4^a,b^** | 1 | chr8: C144516594A | L175L | synonymous SNV | Silent | - | - | - |
| **SMO^a^** | 11 | chr7: GG129209297TT | G456F | nonframeshift substitution | Unknown | - | - | - |
| **TERT** | 7 | chr5: G1293640A | R416* | stopgain | Unknown | - | - | - |

^a^ Gene identified in Everson et al.^1^

^b^ Synonymous SNV without change coded amino acid.

^c^ H1047A variant has not been described. H1047L, H1047R, and H1047Y are classified as pathogenic. The E542 and H1047L, H1047R, and H1047Y variants are classified in OncoKB as 3A for breast, ovarian, and endometrial cancers. Level 3A: **Compelling clinical evidence** supports the biomarker as being predictive of response to a drug **in this indication** but neither biomarker and drug are standard of care

^d^ E17F variant is classified in OncoKB as Therapeutic Level 1 for breast cancer. Level 1: **FDA-recognized** biomarker predictive of response to an **FDA-approved** drug **in this indication**

^e^ Y89C reported to be Likely Oncogenic, Likely Loss-of-function

**Table 6: Patient Characteristics of Tumors for FOXC1 Immunostaining**

| **Sample** | **Age** | **Tissue** | **Grade** |
| --- | --- | --- | --- |
| Dura933 | 90 | Dura | Dura |
| USC-MN8-DURA | 53 | Dura | Dura |
| USC-MN-9-DURA | 62 | Dura | Dura |
| USC-MN-10-DURA | 44 | Dura | Dura |
| MC3 | 47 | Meningioma | I |
| MC4 | 44 | Meningioma | I |
| MC8 | 25 | Meningioma | I |
| USC-MN-10 | 44 | Meningioma | I |
| MC1-S | 39 | Meningioma | II |
| MC6 | 33 | Meningioma | II |
| USC-MN-9 | 62 | Meningioma | II |
| USC-MN-5 | 47 | Meningioma | III |
| USC-MN8 | 53 | Meningioma | III |

Table S1 -DifferentiallyMethylatedProbes.xlsx

Table S2 -DifferentiallyMethylatedPromoters.xlsx

Table S3 -DifferentiallyMethylatedPromoters NF2alt vs Wt.xlsx

Table S4 -DifferentiallyMethylatedPromoters NF2alt and NF2wt vs Dura.xlsx

**Supplementary Figure 1)** **Meningiomas exhibit a low mutational burden**. a-b) Lollipop plots of mutations observed in NF2 (a) and PIK3CA (b). c) OncoPrint of all identified CNVs and mutations. d) Chromosome heatmap of bin copy number assignments with ratio cutoff of 0.25. WHO grade II/III tumors exhibit increased genetic instability. e) OncoPrint of genetic alterations affecting components of the SWI/SNF (BAF) complex. Alterations were detected in 8/10 (80%) WHO grade II/III tumors and 10/21 (47.6%). f) OncoPrint of recurrent genetic alterations alterations. LOH: Loss of heterozygosity.


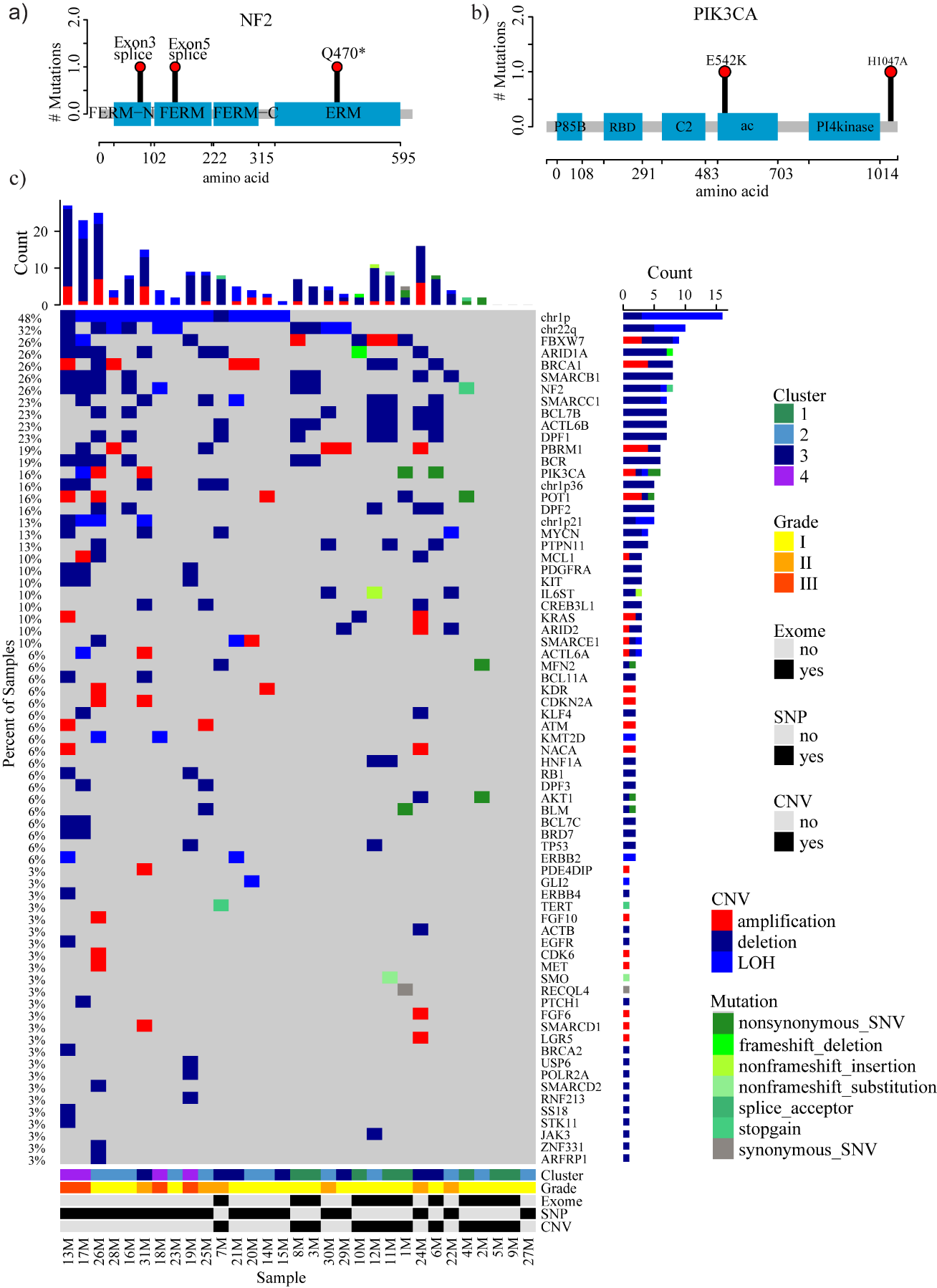


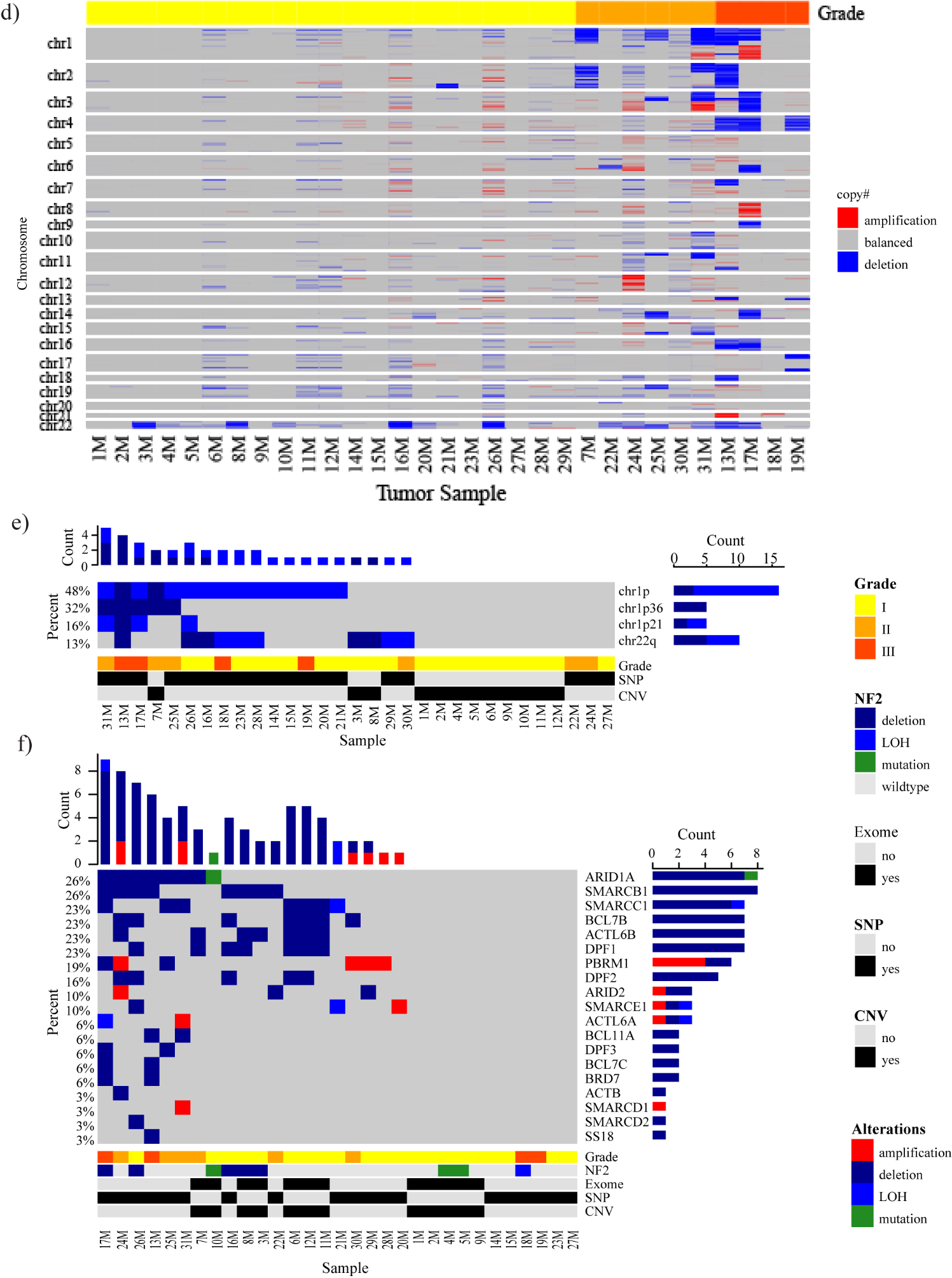


**Supplementary Figure 2)** **Unsupervised Clustering Identifies Methylation Subgroups**

a) Density plot of β-values used to classify probes as unmethylated β<0.3 or methylated (including hemi-methylated) β≥0.3. b) Top: Consensus Cumulative Distribution Function (CSF) plot of the CDF versus the consensus index for κ=2-10 clusters. Bottom: Delta area plot of the change in the area under the consensus cumulative distribution function (CDF) curve versus the number of clusters κ. c) Cluster dendrogram of 4 clusters generated using K-means clustering of Euclidean distance matrix. d) Heatmap showing percentage of meningiomas in each cluster from each of WHO grades I, II, and III. X-axis: WHO grade. Y-axis: Methylation cluster. d) Scree plot showing that 94.38% of variability in probes is attributable to the first principal component. X-axis: principal component. Y-axis: Percent contribution to variability. f) Singular value decomposition analysis of the contribution of sample characteristics to the principal components of variability in the data. g) OncoPrint of altered gene alterations from Figure 1 relative to cluster and grade.


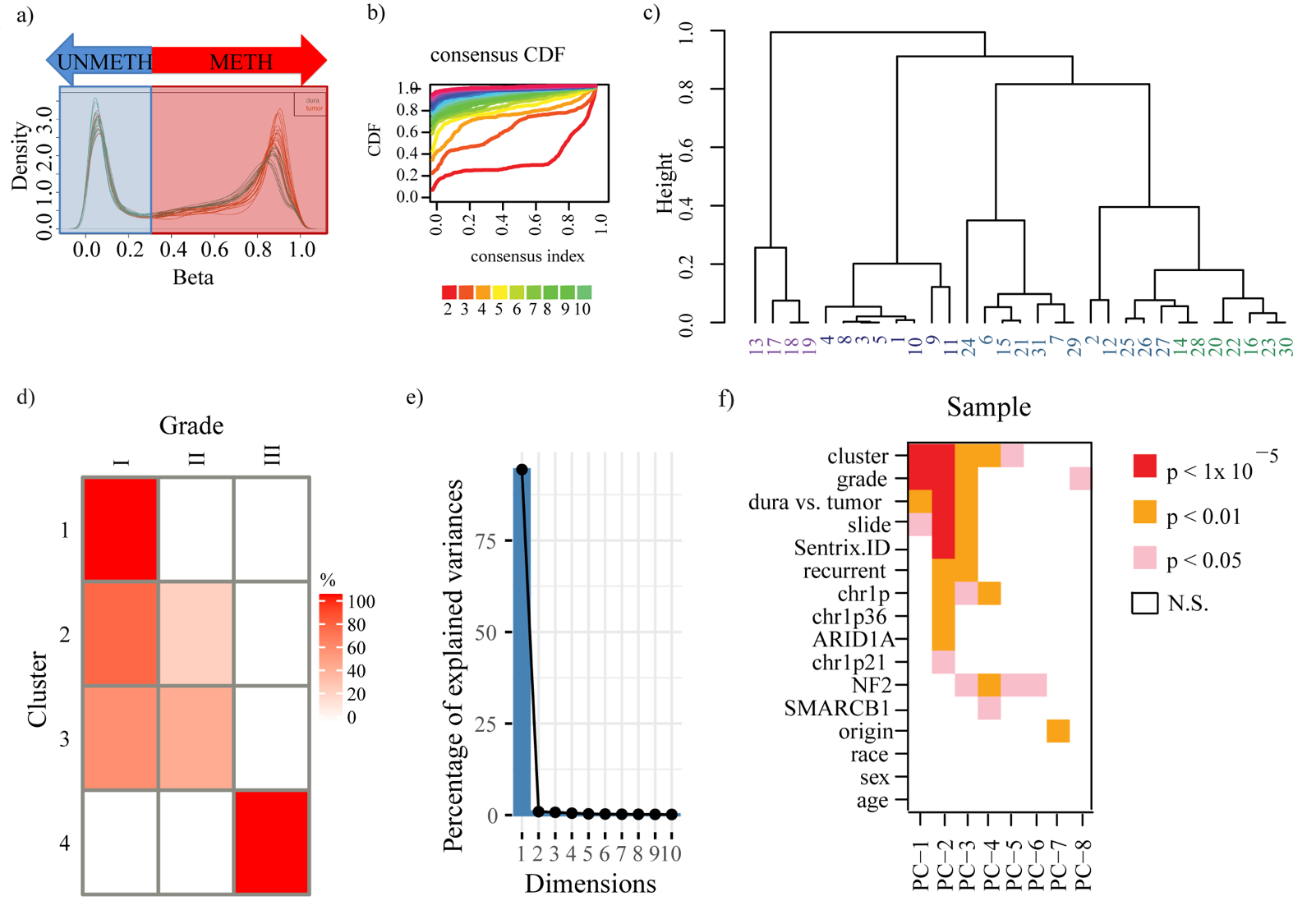


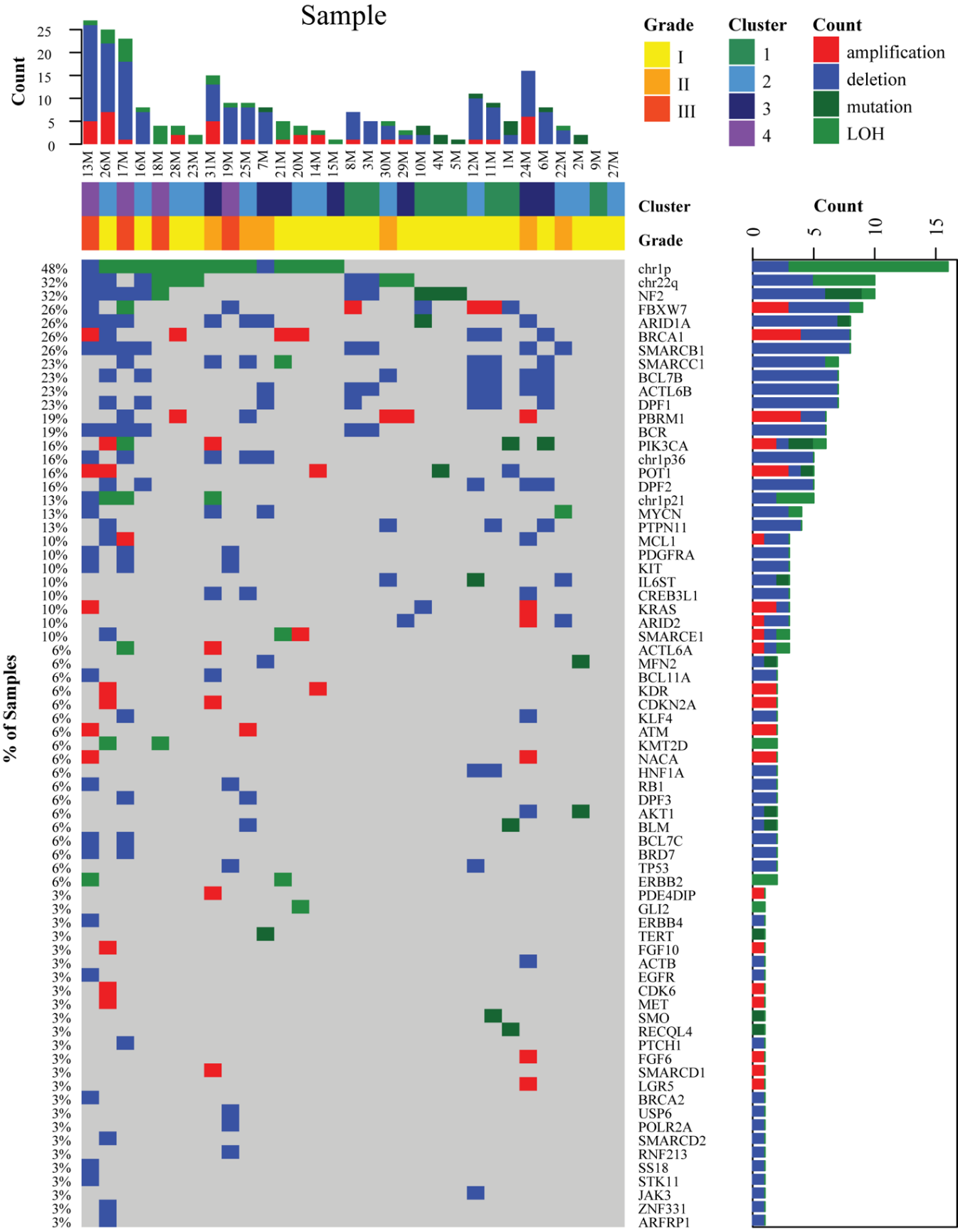


**Supplementary Figure 3) Differential Promoter Methylation.** a-b) IGV plot of β_meningioma_-β_dura_ for the lncRNA *FOXC1 Upstream Transcript (FOXCUT)* and *Bone Morphogenic Protein 4 (BMP4)* for patients 1-4. Differences for each probe are calculated between meningioma and dura from the same patient. c) String interaction plot of the protein interaction network of FOXC1 and BMP4. d) MSigDB GeneOntology terms enriched among significantly hypomethylated genes for each cluster. e) MSigDB curated genesets enriched for each cluster. f-g) IGV plot of meanβ_cluster_-meanβ_dura_ of the *HOXC* cluster (f) and the mesenchymal patterning transcription factor *TBXT (Brachyury)* (g). Red: hypermethylated relative to mean(β_dura_). Blue: hypomethylated relative to mean(β_dura_). h) Barplot from cBioportal query of 16 genes with differentially methylated promoters common to multiple clusters using cancer studies from a standard curated set of 187 cancer studies representing 48,341 samples. x-axis: All cancer types with at least one of the genes altered in >20% of samples. y-axis: Percentage of samples with at least one altered gene in the geneset. i) cBioPortal OncoPlot of alterations for each gene. x-axis: Cumulative sum of alterations. y-axis: Gene.

­­­
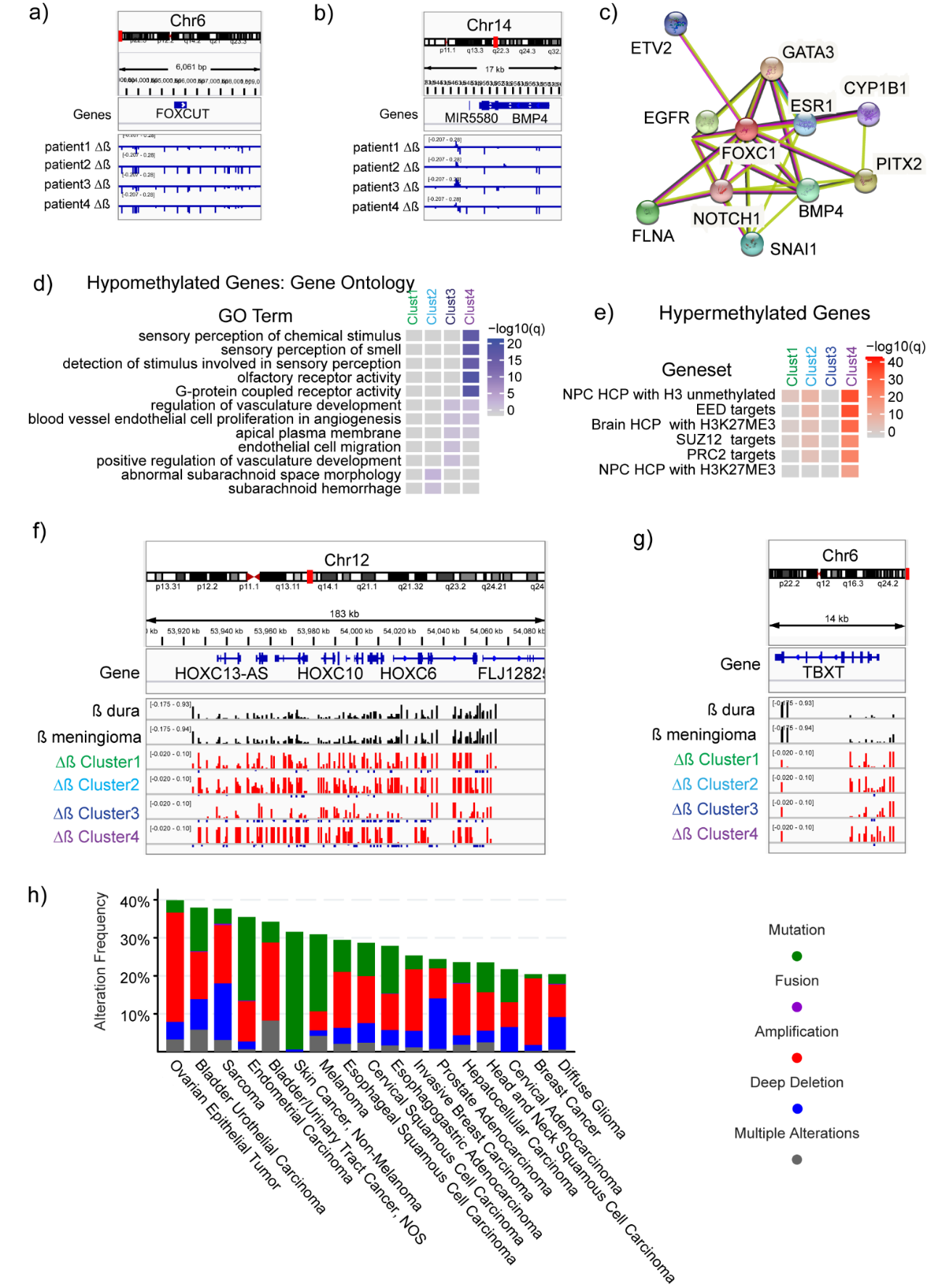


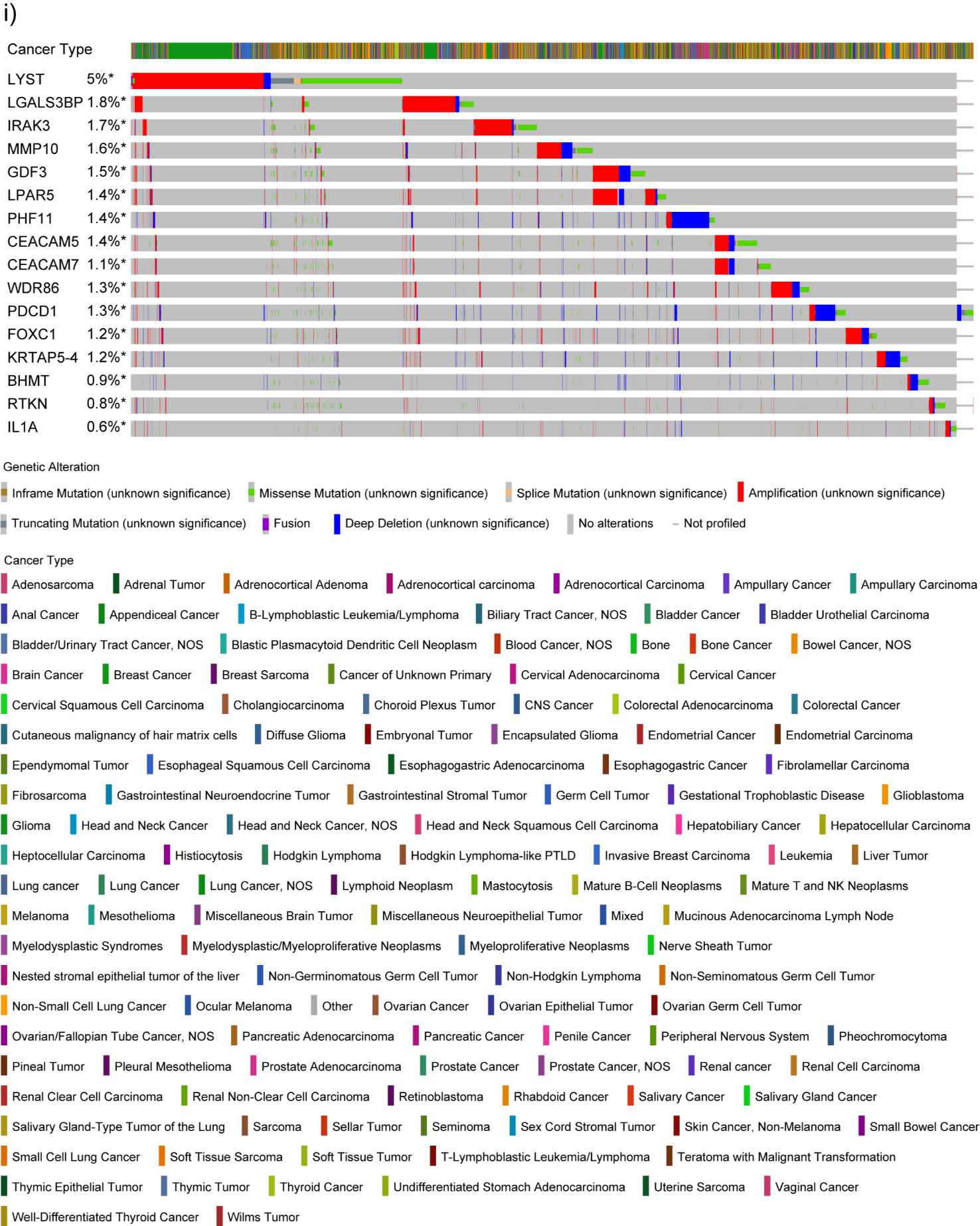


**Supplementary Figure 4) NF2 mutation status and Methylation**. GeneOntology Terms enriched for hypomethylated (a) or hypermethylated (b) gene promoters in *NF2* altered meningiomas when compared to *NF2* wildtype meningiomas. x-axis: Gene Count. y-axis: GeneOntology term. Legend: adjusted p-value (Benjamini-Hochberg). c) Coronal image from patient 4. Thickening of dura extends beyond the margins of the craniotomy sites. Large grey arrows: edges of craniotomy. Small white arrows: Thickened dural tail.


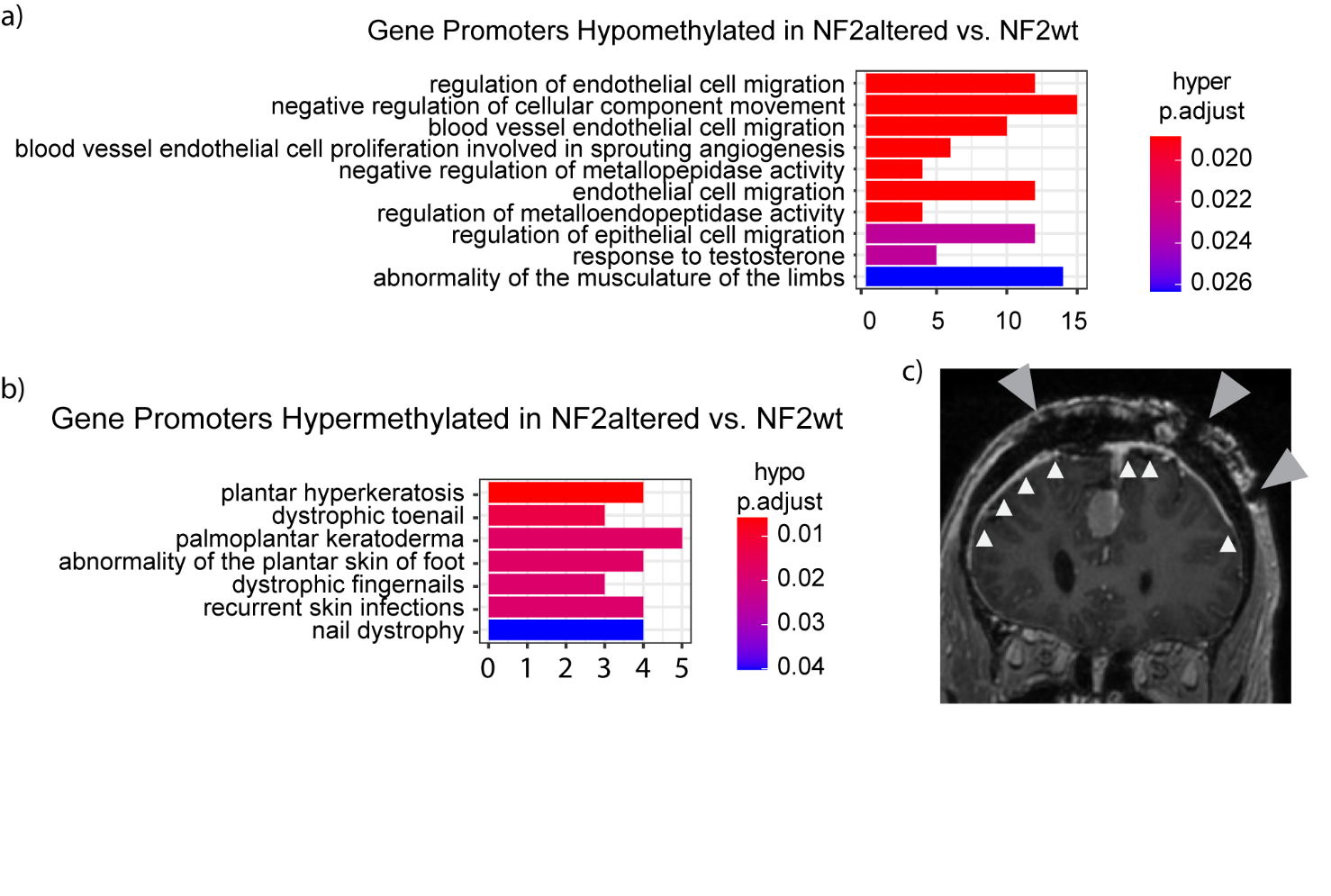

Supplement: vdac084_suppl_Supplementary_Material [file vdac084_suppl_supplementary_material.docx]
